# Characterization of a novel panel of plasma microRNAs that discriminates between *Mycobacterium tuberculosis* infection and healthy individuals

**DOI:** 10.1371/journal.pone.0184113

**Published:** 2017-09-14

**Authors:** Jia-Yi Cui, Hong-Wei Liang, Xin-Ling Pan, Di Li, Na Jiao, Yan-Hong Liu, Jin Fu, Xiao-Yu He, Gao-Xiang Sun, Chun-Lei Zhang, Chi-Hao Zhao, Dong-Hai Li, En-Yu Dai, Ke Zen, Feng-Min Zhang, Chen-Yu Zhang, Xi Chen, Hong Ling

**Affiliations:** 1 Department of Microbiology, Harbin Medical University, Harbin, China; 2 Heilongjiang Provincial Key Laboratory of Infection and Immunity; Key Laboratory of Pathogen Biology, Harbin, China; 3 Wu Lien-Teh Institute, Harbin Medical University, Harbin, China; 4 State Key Laboratory of Pharmaceutical Biotechnology, NJU Advanced Institute for Life Sciences (NAILS), Jiangsu Engineering Research Center for MicroRNA Biology and Biotechnology, School of Life Sciences, Nanjing University, Nanjing, China; 5 Harbin Chest Hospital, Harbin, China; 6 Department of Laboratory Medicine, The Second Affiliated Hospital of Harbin Medical University, Harbin, China; 7 Department of Neurology, The Second Affiliated Hospital of Harbin Medical University, Harbin, China; 8 Department of Parasitology, Harbin Medical University, Harbin, China; Huazhong University of Science and Technology, CHINA

## Abstract

Cavities are important in clinical diagnosis of pulmonary tuberculosis (TB) infected by *Mycobacterium tuberculosis*. Although microRNAs (miRNAs) play a vital role in the regulation of inflammation, the relation between plasma miRNA and pulmonary tuberculosis with cavity remains unknown. In this study, plasma samples were derived from 89 cavitary pulmonary tuberculosis (CP-TB) patients, 89 non-cavitary pulmonary tuberculosis (NCP-TB) patients and 95 healthy controls. Groups were matched for age and gender. In the screening phase, Illumina high-throughput sequencing technology was employed to analyze miRNA profiles in plasma samples pooled from CP-TB patients, NCP-TB patients and healthy controls. During the training and verification phases, quantitative RT-PCR (qRT-PCR) was conducted to verify the differential expression of selected miRNAs among groups. Illumina high-throughput sequencing identified 29 differentially expressed plasma miRNAs in TB patients when compared to healthy controls. Furthermore, qRT-PCR analysis validated miR-769-5p, miR-320a and miR-22-3p as miRNAs that were differently present between TB patients and healthy controls. ROC curve analysis revealed that the potential of these 3 miRNAs to distinguish TB patients from healthy controls was high, with the area under the ROC curve (AUC) ranged from 0.692 to 0.970. Moreover, miR-320a levels were decreased in drug-resistant TB patients than pan-susceptible TB patients (AUC = 0.882). In conclusion, we identified miR-769-5p, miR-320a and miR-22-3p as potential blood-based biomarkers for TB. In addition, miR-320a may represent a biomarker for drug-resistant TB.

## Introduction

According to the Global Tuberculosis Report 2016 by World Health Organization (WHO), the tuberculosis (TB) epidemic is higher than previously estimated [1]. China is still in the list of the six highest TB burden countries, and the cases in the six countries accounts for 60% of new TB cases. In 2015, the Sustainable Development Goals (SDGs) for 2030 were adopted by the United Nations and one of their main goals was to end the global TB epidemic [1]. Since 2000, the incidence of TB dropped by an average of 1.5% per year worldwide. However, to reach the first milestone of the “End TB Strategy”, it is essential that, by 2020, the minimum annual decline should be 4–5%. In countries with a high TB burden, the trend of multidrug-resistant TB or rifampin-resistant TB (MDR-TB/RR-TB) and mortality decline are similar to incidence. Thus, there is still a long way to go to meet the targets of the SDGs.

The spreading of *Mycobacterium tuberculosis* (*M*. *tuberculosis*) from both active TB patients and TB cases with cavity causes an uncontrolled epidemic of TB and drug-resistant TB. The pathology, pathogenesis and cavitation of TB have been extensively studied. However, the underlying mechanisms of action remain to be elucidated. *M*. *tuberculosis* modulates inflammation at distinct stages of life. Cavitation, as a result of hyper-inflammatory tissue-damaging events, is derived by the formation of granulomas and is associated with disease progression and transmission [2]. Cavitary lesions, which are rich in *M*. *tuberculosis*, contain a thin caseous-necrotic layer [3, 4]. Previous studies suggested a correlation between cavities in active TB patients and high levels of bacilli in sputum [5, 6]. In addition, cavities impair the efficacy of antimicrobials and may therefore increase the risk of antibiotic resistance and result in failure of treatment [2].

It is well recognized that, during mycobacterial infections, microRNAs (miRNAs) emerge as important regulators of the immune response [7]. miRNAs were differentially regulated upon mycobacterial infection of macrophages, both *in vitro* and *in vivo*. Bacterial cell-wall components from virulent mycobacterial species induce differential expression of miRNAs in infected macrophages [8]. Lipomannan from *M*. *tuberculosis* or *M*. *smegmatis* induced the expression of miR-125b and miR-155 *in vitro* [9]. Furthermore, miR-125b directly targeted TNF-α, whereas miR-155 affected the PI3K/Akt pathway by modulating the function of SHIP1 [9]. The cytokines IFN-γ and TNF-α are key mediators in protecting immunity in TB and are involved in modulating the recruitment of inflammatory leukocytes to the lungs.

MiRNAs are essential in a wide array of biological processes and could serve as novel biomarkers for the diagnosis, treatment monitoring and prognosis of a broad range of diseases including TB [10–15]. In the circulation, plasma miRNAs are stable and protected from endogenous RNase activity. In fact, circulating miRNA levels are consistent among individuals [16].

In this study, we investigated miRNA expression profiles in plasma samples from pulmonary TB patients (with or without cavities) and compared this with miRNA levels from healthy controls. We aimed to identify plasma miRNAs that are associated with pulmonary TB as well as with cavity status.

## Materials and methods

### Patients and control subjects

A total of 273 participants, including 178 patients who were diagnosed with pulmonary TB in the Harbin Chest Hospital (Harbin, China) and 95 healthy subjects were recruited from various districts in the Heilongjiang Province (China) between June 2011 and March 2013. Blood samples were collected at the patients’ first admission to the hospital. None of the patients were diagnosed with diabetes, hepatitis B, immune deficiency disease or other pulmonary-associated diseases. Patient characteristics are summarized in Table 1. Control participants were recruited from a large pool of individuals who underwent a routine health checkup at the Second Affiliated Hospital of Harbin Medical University. Individuals who showed no evidence of disease were selected as healthy controls. Patients and controls were matched based on age and gender. All the participants provided their written informed consent to participate in the study and the protocol was approved by the Institutional Research Board of the University of Harbin Medical University (Harbin, China).

**Table 1:** Demographic and clinical characteristics of CP-TB patients, NCP-TB patients and healthy individuals in training and validation sets.

Variable	CP-TB (n = 64)	NCP-TB (n = 64)	Healthy control (n = 64)
Age, years^**a**^	43.4 (18.84)	43.3 (18.26)	42.3 (17.41)
Age, group, n			
≤25	16 (25%)	14 (21.9%)	13 (20.3%)
26–40	15 (23.4%)	17 (26.6%)	18 (28.1%)
41–55	18 (28.1%)	16 (25%)	19 (29.7%)
≥56	15 (23.4%)	17 (26.6%)	14 (21.9%)
Sex, n			
Male	44 (68.8%)	41 (64.1%)	34 (53.1%)
Female	20 (31.2%)	23 (35.9%)	30 (46.9%)
History of TB treatment			
Yes	16 (25%)	17 (26.6%)	
No	48 (75%)	47 (73.4%)	

^a^ Age data are presented as the mean (SD).

Abbreviations: CP-TB: cavitary pulmonary tuberculosis; NCP-TB: non-cavitary pulmonary tuberculosis; TB: tuberculosis

**Fig 1:**
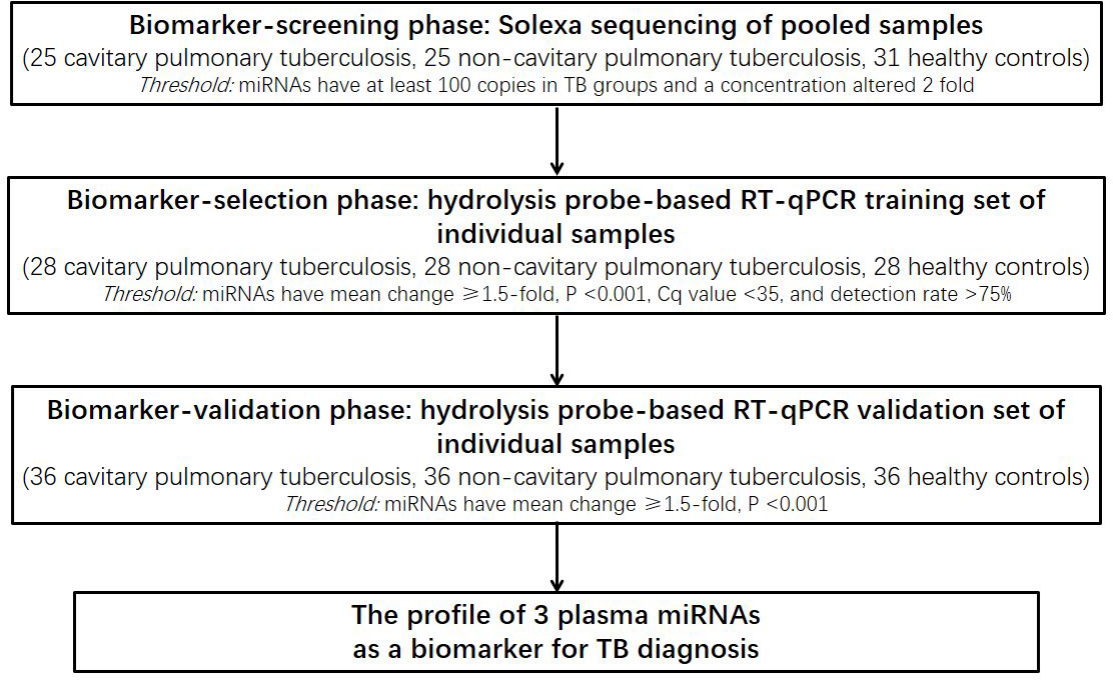
Flow chart of the experimental design.

A multiphase, case-control study was conducted to identify miRNAs in plasma as surrogate markers for TB (Fig 1). To identify miRNAs that show differential expression between TB cases and matched controls, an initial biomarker screening was performed. For this screening, pooled plasma samples from 25 cavitary pulmonary tuberculosis (CP-TB) patients, 25 non-cavitary pulmonary tuberculosis (NCP-TB) patients and 31 healthy controls, underwent Illumina high-throughput sequencing (miRBase 12.0; total, 692 miRNAs). Subsequently, a biomarker confirmation analysis, to refine the plasma miRNA levels as the TB signature, was performed by using a hydrolysis probe-based qRT-PCR assay. This analysis was performed in 2 phases: (a) the biomarker-selection phase, in which plasma samples from 28 CP-TB patients, 28 NCP-TB patients and 28 healthy controls served as the training set, and (b) the biomarker-validation phase, in which plasma samples from additional 36 CP-TB patients, 36 NCP-TB patients and 36 healthy controls served as the validation set.

### RNA extraction

From each patient, venous blood samples (approximately 5 mL) were collected in sodium citrate coated tubes. After overnight incubation at 4°C, plasma was collected and stored at -80°C for future analysis.

For Illumina high-throughput sequencing, equal volumes of plasma from 25 CP-TB patients and 25 NCP-TB patients (400 μL per subject) and from 31 matched healthy controls (322 μL per subject) were pooled to form the case and control sample pools. To extract total RNA from each pool of plasma samples, TRIzol reagent (Invitrogen, Carlsbad, CA, USA) was used as previously described [17]. The resulting RNA pellet was dissolved in 30 μL diethyl pyrocarbonate-treated (DEPC-treated) water and stored at -80°C for future analysis. For qRT-PCR assays, total RNA was extracted using a one-step acid phenol/chloroform purification as described in previous study [17]. The pellet was dissolved in 20 μL of DEPC water and stored at -80°C until further analysis.

### Illumina high-throughput sequencing

The small RNA molecules (< 30 bases) were purified by PAGE and a pair of high-throughput sequencing adaptors were ligated to the 5′ and 3′ ends, then small RNA molecules were amplified for 17 cycles using adaptor primers. Fragments of 90 bp (small RNA+adaptors) were purified from an agarose gel. Purified DNA was used for cluster generation and sequencing analysis by Illumina high-throughput sequencing according to the manufacturer’s instructions. The data and results were generated as previously described [18]. Clean reads were compared using a miRBase database (release 20.0). The total copy number of each sample was normalized to 100,000.

### Quantification of miRNAs by quantitative RT-PCR (qRT-PCR)

Hydrolysis probe–based qRT-PCR was performed according to the manufacturer’s instructions (LightCycler® 480 II Instrument, Roche) with minor modifications. The reverse transcription was carried as previously described [17]. For cDNA synthesis, reaction mixtures were incubated at 16 ^o^C for 15 min, at 42 ^o^C for 1 h, at 85 ^o^C for 5 min, and held at 4 ^o^C. The qRT-PCR was the same as previously described [17]. All experiments, including no-template controls, were carried out in triplicate. A combination of let-7d, let-7g and let-7i (let-7d/g/i) were served as an endogenous control for normalizing qRT-PCR data, and the detailed information was previously described [17, 19]. Relative levels of miRNAs were normalized to let-7d/g/i and were calculated using the 2^-ΔΔCq^ method [17, 20].

### Statistical analysis

Statistical analyses were performed by using the Statistical Analysis System software SPSS 16.0. Data were displayed as the mean ± SD. The differences between groups were compared by using the Student’s *t*-test or two-sided χ^2^ test. The statistically significances were defined as a *P-*value of <0.05. Receiver-operating-characteristic (ROC) curves and the area under the ROC curves (AUC) were constructed to evaluate the predictive power of candidate miRNAs for CP-TB, NCP-TB and TB. To evaluate the association between plasma miRNAs levels and TB, risk score analysis was performed as previously described [21]. Briefly, the risk score of each miRNA, denoted as s, was set to 1 if the expression level was less than the lower 5% reference interval of the corresponding miRNA level in healthy controls. In the case of an expression level above 5%, the risk score was set to 0. A risk score function (RSF), to predict TB, was defined according to a linear combination of the expression level for each miRNA using the followed equation [17].:

$$rsf_{i}=\sum_{j=1}^{n}W_{j}\cdot s_{ij}$$ (1)

In the Eq (1), the risk score for miRNA *j* on sample *i* expressed in *s*_*ij*_. and it’s weight of the risk score expressed in W_*j*_.We fit *n* univariate logistic regression models using the disease status with each of the risk scores To determine the *W·s*. And then we use the regression coefficient of each of the risk scores as the weight to indicate each miRNA’s contribution to the RSF. Samples were ranked according to their RSF and then divided into the following two groups: 1) a high-risk group, representing the predicted TB cases, and 2) a low-risk group, representing the predicted controls. Frequency tables and ROC curves were used to evaluate diagnostic profiling effects and to elucidate the appropriate cut-off point.

## Results

### Profiling plasma miRNAs in TB patients using high-throughput sequencing

**Fig 2:**
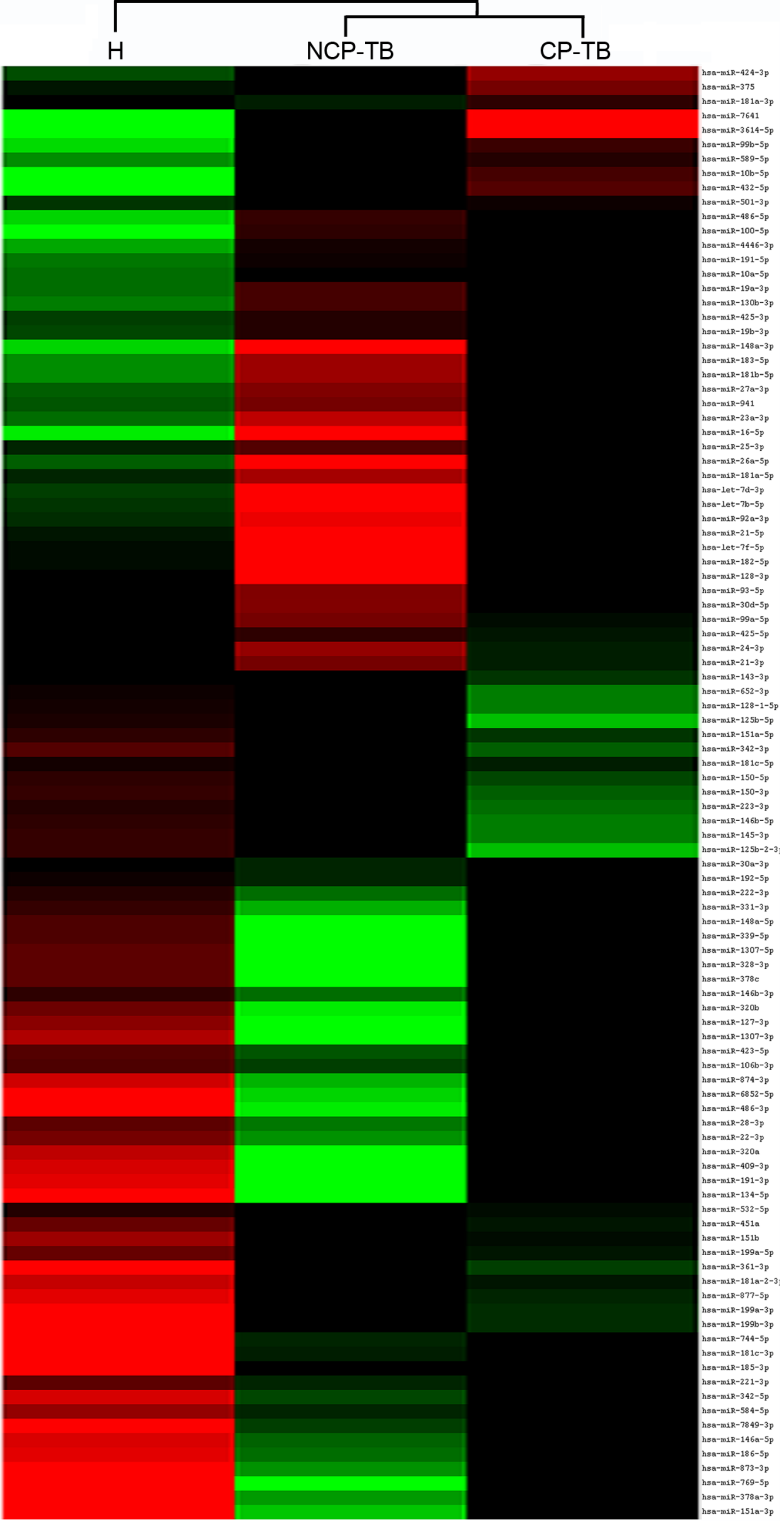
MiRNA expression in plasma derived from CP-TB, NCP-TB patients and healthy controls (H).

Initially, expression profiles of plasma miRNAs were screened to identify significantly altered miRNAs between TB patients and healthy controls. Total RNA was extracted from healthy controls (plasma derived from 31 individuals was pooled), CP-TB patients (plasma derived from 25 individuals was pooled) and NCP-TB patients (plasma derived from 25 individuals was pooled). Equal amounts of total RNA were analyzed through Illumina high-throughput sequencing. As a result, a total of 2,220,577; 2,230,331 and 2,768,917 reads of RNAs ranging from 18 to 30 nucleotides were obtained from pooled plasma samples of healthy controls, CP-TB patients and NCP-TB patients, respectively. The three pools of plasma samples contained various length of small RNAs (S1 Fig). Then bioinformatics tools were employed to investigate small RNA species and sequencing frequencies. In plasma derived from TB patients and healthy controls, multiple and heterogeneous small RNA species, including miRNAs, piRNAs, rRNAs and tRNAs were identified (S1 Table). We found that in plasma samples of TB patients and healthy controls, miRNAs occupied roughly 20% of the total amount of small RNAs (sequencing reads). A total of 297, 427 and 280 miRNAs were identified in healthy controls, CP-TB patients and NCP-TB patients, respectively (Fig 2). In addition, significant alterations were observed in plasma miRNA profiles from CP-TB and NCP-TB patients compared with healthy controls (S2 Fig).

To further narrow down the list of plasma miRNAs as TB biomarkers, we applied the criteria for including plasma miRNAs as follows: for CP-TB and NCP-TB patients compared with healthy controls, sequencing reads should be larger than 500 and there should be at least a 4-fold difference in miRNA expression between the comparative groups. Consequently, 29 plasma miRNAs met the inclusion criteria (S2 Table). Moreover, we included another miRNA, miR-22-3p, as a candidate, since this miRNA has been previously reported to be upregulated in serum of TB patients [22]. However, because the qRT-PCR probes for 3 (miR-6852-5p, miR-1307-3p and miR-1307-5p) of the 30 candidate miRNAs were currently unavailable, a final list of 27 plasma miRNAs was chosen for further analysis (S3 Table).

### Selection of significantly altered plasma miRNAs between TB patients and healthy controls

To identify differentially expressed plasma miRNAs as a TB fingerprint, the candidate miRNAs (*n* = 27) underwent TaqMan probe-based qRT-PCR analysis, for which two sets of individual plasma samples from 64 healthy controls and 128 TB patients were used. All patients enrolled in the study (*n* = 128) were clinically and pathologically diagnosed by sputum bacteria cultures and X-ray analysis. No significant differences were observed in demographic characteristics between TB patients and healthy controls (Table 1).

Initially, the 27 candidate miRNAs were measured in the training set including 28 healthy controls, 28 CP-TB patients and 28 NCP-TB patients. In this phase, we only focused on miRNAs that showed a *P*-value < 0.005 between any patients group and controls. Using these criteria, a list of 7 miRNAs (miR-769-5p, miR-320a, miR-22-3p, miR-151a-3p, miR-103a-3p, miR-107 and miR-148a-3p) was generated (Table 2). Among these miRNAs, levels of miR-769-5p, miR-320a, miR-22-3p and miR-151a-3p were significantly decreased in TB patients compared with healthy controls. In contrast, levels of miR-103a-3p, miR-107 and miR-148a-3p were increased in patients compared with controls. Between the NCP-TB and CP-TB patients, only miR-320a showed a significant alteration (*P* = 0.034).

**Table 2:** Relative miRNA expression level to let-7 in plasma samples derived from TB patients and control subjects in the training set.

MiRNA	H (*n* = 28)	NCP-TB (*n* = 28)	-Fold change (H/NCP-TB)	*P*	CP-TB (*n* = 28)	-Fold change (H/CP-TB)	*P*	-Fold change (CP-TB/NCP-TB)	*P*
miR-769-5p	11.34 (10.21)	3.44 (2.52)	3.30	< 0.001	4.30 (3.74)	2.64	0.001	1.25	0.324
miR-22-3p	0.68 (0.08)	0.33 (0.07)	2.06	0.002	0.41 (0.08)	1.66	0.017	1.24	0.403
miR-320a	476.39 (43.93)	220.24 (27.56)	2.16	< 0.001	375.71 (45.68)	1.27	0.225	1.71	0.034
miR-151a-3p	1.27 (0.13)	0.67 (0.08)	1.90	< 0.001	0.83 (0.13)	1.53	0.031	1.24	0.287
miR-103a-3p	0.20 (0.04)	0.34 (0.05)	0.59	0.034	0.42 (0.05)	0.48	0.001	1.24	0.282
miR-107	0.05 (0.01)	0.10 (0.01)	0.50	0.002	0.11 (0.01)	0.45	< 0.001	1.10	0.606
miR-148a-3p	0.05 (0.01)	0.09 (0.01)	0.56	0.006	0.09 (0.01)	0.56	0.002	1.00	0.855

Abbreviations: H: healthy controls; CP-TB: cavitary pulmonary tuberculosis; NCP-TB: non-cavitary pulmonary tuberculosis

**Fig 3:**
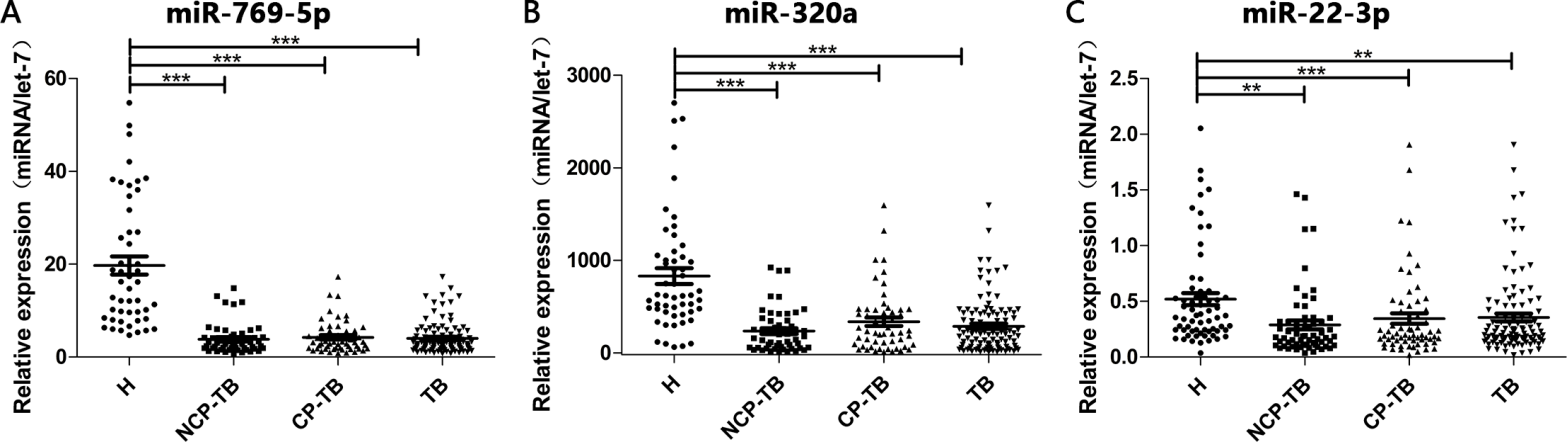
Detection of TB using 3 plasma miRNAs as a biomarker. A hydrolysis probe–based qRT-PCR assay was used to measure the relative levels of the 3 miRNAs in 64 CP-TB patients, 64 NCP-TB patients and 64 healthy controls (in both the training and validation set). Each point represents the mean of the results for triplicate. The asterisks indicate significant differences compared to healthy controls. * *P*<0.05; ** *P*<0.01; *** *P*<0.001. (A) miR-769-5p, (B) miR-320a and (C) miR-22-3p.

To confirm the accuracy and specificity of the 7 miRNAs as a TB signature, their expression levels were further assessed in an independent larger cohort (validation set), which contained 36 healthy controls, 36 CP-TB patients and 36 NCP-TB patients. The alteration of 3 plasma miRNAs (miR-769-5p, miR-320a and miR-22-3p) was consistent between the training set and the validation set (Tables 2 and 3). The differential expression levels of miR-769-5p, miR-320a and miR-22-3p between plasma samples of TB patients and healthy controls was shown in Fig 3. In summary, a profile of 3 plasma miRNAs was selected as a potential signature for TB.

**Table 3:** Relative miRNA expression level to let-7 in plasma samples derived from TB patients and control subjects in the validation set.

miRNA	H (*n* = 36)	NCP-TB (*n* = 36)	-Fold change (H/NCP-TB)	*P*	CP-TB (*n* = 36)	-Fold change (H/CP-TB)	*P*	-Fold change (CP-TB/NCP-TB)	*P*
miR-769-5p	36.89 (3.43)	1.41 (0.21)	26.16	< 0.001	1.44 (0.17)	25.62	< 0.001	1.02	0.920
miR-22-3p	0.31 (0.02)	0.21 (0.02)	1.48	0.002	0.20 (0.02)	1.55	< 0.001	0.95	0.720
miR-320a	850.59 (111.15)	212.70 (40.31)	4.00	< 0.001	264.42 (52.45)	3.22	< 0.001	1.24	0.448

Abbreviations: H: healthy controls; CP-TB: cavitary pulmonary tuberculosis; NCP-TB: non-cavitary pulmonary tuberculosis

### Discrimination accuracy of the selected 3 plasma miRNAs as a TB fingerprint

**Fig 4:**
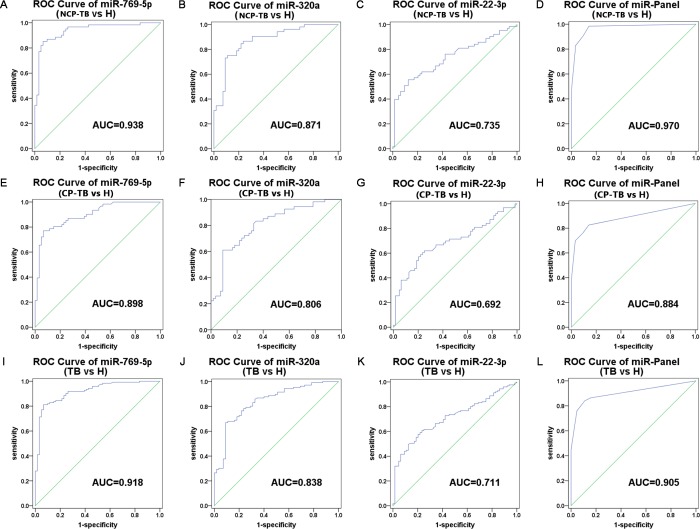
ROC curves to compare the ability of miRNA to distinguish TB patients from the healthy controls. (A-D) miR-769-5p, miR-320a, miR-22-3p and the three-miRNA panel for discriminating between NCP-TB patients and healthy controls; (E-H) miR-769-5p, miR-320a, miR-22-3p and the three-miRNA panel for discriminating between CP-TB patients and healthy controls; (I-L) miR-769-5p, miR-320a, miR-22-3p and the three-miRNA panel for discriminating between TB patients and healthy controls.

To evaluate the selected plasma miRNAs in discriminating between TB patients and healthy controls, the ROC curve analysis was conducted by using the entire sample set. ROC curve analysis demonstrated that miR-769-5p, miR-320a and miR-22-3p can serve as potential biomarkers for discriminating NCP-TB patients from healthy controls, with AUC being 0.938, 0.871 and 0.735, respectively (Fig 4A–4D). Likewise, the miR-769-5p, miR-320a and miR-22-3p discriminated CP-TB patients from healthy controls, with AUC being 0.898, 0.806 and 0.692, respectively (Fig 4E–4H). Moreover, when ROC curves were analyzed in combination of CP-TB and NCP-TB patients, miR-769-5p, miR-320a and miR-22-3p could distinguish TB patients from healthy controls, with AUC being 0.918, 0.838 and 0.711, respectively (Fig 4I–4L). On the other hand, miR-769-5p, miR-320a and miR-22-3p could not distinguish CP-TB patients from NCP-TB patients. The above results indicated that miR-769-5p, miR-320a and miR-22-3p can be useful in distinguishing TB patients from healthy controls, but the differences between CP-TB and NCP-TB patients are not significant.

### Distinguishing TB patients from healthy controls by using risk core analysis

To further evaluate the potential miRNA signature in distinguishing TB patients from healthy controls, a risk score analysis was performed. First, in the training set, the risk score equation was used to define all samples as a high-risk group (representing predicted TB patients), or a low-risk group (representing predicted healthy controls) based on an optimal cutoff value (the value of sensitivity + specificity is maximal) [18]. At the cutoff value of 2.014, only 4 healthy controls in the training set showed a risk score > 2.014, and 50 out of the 56 TB patients exhibited a risk score > 2.014 (Table 4). Using the risk score formula with the same cutoff value in the validation setout of 72 TB patients and 36 healthy controls, 15 patients and 5 healthy controls were incorrectly predicted by the scoring method (Table 4). In addition, we integrated the 3-miRNA signature into a single biomarker using the risk score function and evaluated the accuracy of the miRNA signatures for discriminating TB patients from healthy controls. A combined AUC value of 0.905 was obtained (Fig 4L). These results suggested a strong correlation between plasma miRNA expression and disease state in TB patients.

**Table 4:** Risk score analysis of TB patients and healthy controls.

Score	0–2.014	>2.014	PPV^a^	NPV^b^
Training set			0.926	0.800
Healthy controls	24	4		
TB	6	50		
Validation set			0.919	0.674
Healthy controls	31	5		
TB	15	57		

^a^PPV, positive predictive value

^b^NPV, negative predictive value; TB, tuberculosis.

### Significantly altered plasma miRNAs between drug-resistant TB patients and pan-susceptible TB patients

**Fig 5:**
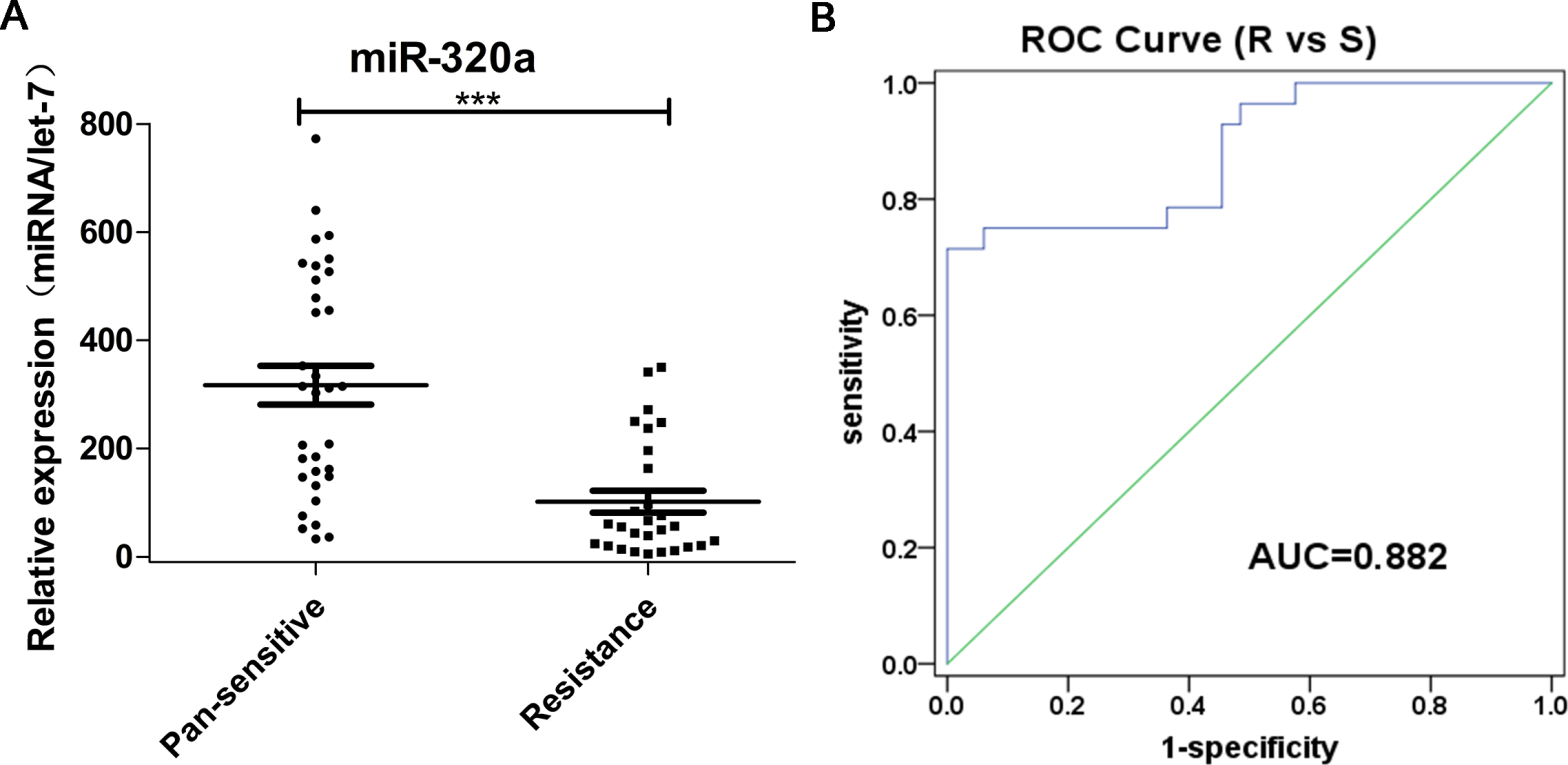
Downregulation of miR-320a in plasma from drug-resistant TB patients compared with drug-susceptible TB patients. (A) Relative concentration of miR-320a in plasma derived from drug-resistant and drug-susceptible TB patients. (B) ROC curves to comparing the ability of miR-320a to distinguish between drug-resistant TB and drug-susceptible TB.

Among the 128 TB patients, we further analyzed 61 TB cases whose first line drug resistance information could be extracted from their clinical records (Table 5). A total of 28 patients were resistant to at least one drug, 10 of which were multidrug-resistant (MDR) TB. We further analyzed if miR-769-5p, miR-320a and miR-22-3p could distinguish drug-resistant TB patients from pan-susceptible TB patients. Levels of miR-320a were decreased in the drug-resistant group (Fig 5A), whereas levels of miR-769-5p and miR-22-3p were comparable between groups (Table 6). In addition, the accuracy of miR-320a for discriminating drug-resistant patients was relatively high, with an AUC value of 0.882 (Fig 5B).

**Table 5:** Demographic and clinical characteristics of drug-resistant TB patients.

Variable	Drug Resistant TB (*n* = 28)	Drug Susceptible TB (*n* = 33)	*P*
Age, years^a^	44.3 (20.29)	44.7 (21.09)	0.94^b^
Age, group, *n*			0.46^b^
≤25	7 (25%)	10 (30.3%)	
26–40	5 (17.9%)	5 (15.2%)	
41–55	10 (35.7%)	8 (24.2%)	
≥56	6 (21.4%)	10 (30.3%)	
Sex, *n*			0.46^c^
Male	17 (60.7%)	23 (69.7%)	
Female	11 (39.3%)	10 (30.3%)	
History of TB treatment			< 0.001
Yes	12 (42.9%)	5 (15.2%)	
No	16 (57.1%)	28 (84.8%)	
Cavity visible on radiograph			0.05^c^
Yes	15 (53.6%)	18 (54.5%)	
No	13 (46.4%)	15 (45.5%)	

^a^ Age data are presented as the mean (SD).

^b^ Student t-test.

^c^ Two-sided 2 test.

**Table 6:** Relative miRNA expression levels to let-7 in plasma samples derived from drug-resistant and pan-susceptible TB patients.

miRNA	Drug-resistant TB (n = 28)	Pan-susceptible TB (n = 33)	-Fold change (drug-resistant TB / pan-susceptible TB)	*P*
miR-769-5p	4.18 (2.26)	5.17 (2.75)	0.81	0.562
miR-22-3p	0.26 (0.48)	0.46 (0.78)	0.57	0.117
miR-320a	101.61 (10.39)	317.08 (14.37)	0.32	< 0.001

## Discussion

In the present study, we examined and validated the plasma miRNA profile in TB patients (including CP-TB and NCP-TB patients) and healthy controls. We found a novel panel of three miRNAs (miR-769-5p, miR-320a and miR-22-3p) that clearly differentiated TB patients from healthy controls, indicating that these miRNAs may serve as potential biomarkers for active TB. In addition, we demonstrated that miR-320a distinguished drug-resistant TB from drug-susceptible TB patients. None of the miRNAs validated by qRT-PCR discriminated between cavity and non-cavity TB conditions.

Numerous miRNAs have found to be stable in plasma and serum [8, 23, 24]. In serum samples of pulmonary TB patients (including active TB patients), the expression levels of multiple miRNAs were significantly upregulated compared with those in healthy controls. Upregulated miRNAs included miR-183, miR-378, miR-483-5p, miR-22, miR-29c, miR-361-5p, miR-889, miR-576-3p, miR-210, miR-26a, miR-432, miR-155, miR-155*, miR-125a, miR-30a, miR-21, miR-582-5p, miR-223, miR-125b, miR-99b, miR-132 and miR-134 [22, 25–27]. Expression levels of miR-101, miR-17-5p, let-7f and miR-320b were significantly downregulated in patients versus healthy controls [8, 22, 25, 26, 28]. In peripheral blood mononuclear cell (PBMC) culture supernatants of active pulmonary TB patients, levels of miR-21*, miR-223, miR-302a, miR-424, miR-451 and miR-486-5p were significantly upregulated compared with latent TB patients. Moreover, miR-130b* levels were significantly downregulated [29]. In addition, expression levels of miR-130a*, miR-296-5p, miR-493*, miR-520d-3p and miR-661 were significantly higher in PBMC culture supernatants of latent TB patients compared with that of healthy controls [29].

The changes in miRNA expression profiles reflect universal responses to mycobacterial pathogens and indicate that miRNAs may be unique and potential biomarkers for TB. The expression profiles of miRNAs indicate the unique characteristics of the disease and reflect different disease stages. Based on these findings, miRNAs alone or in combination have the potential to discriminate between TB patients and healthy controls and may serve as novel biomarkers. A unique miRNA or miRNA pattern used to specifically classify TB patients has not yet been identified.

Contrary to previous findings [22, 30, 31], we found that in TB patients, the expression level of miR-22 was decreased compared to that in healthy controls. In one study, the data was verified by qRT-PCR and showed a limited discrimination ability of miR-22 (AUC = 0.711). Possible reasons for this alteration remain unclear. Recently, it was found that miRNA expression varies and that this is independent of the disease state but, instead relates to geographical or potentially ethnic differences between the cohorts [32]. The significance of the downregulation of miR-22 in plasma of TB patients still needs to be investigated. Furthermore, primary Illumina high-throughput sequencing results showed that in patients a downregulation of plasma miR-320a and miR-769-5p was found, however, these findings have not yet been validated [22, 30, 31]. To our knowledge, we were the first to validate the expression profile of miR-320a and miR-769-5p in TB patients. In addition, we identified that a combination of three miRNAs including miR-22, miR-320a, and miR-769-5p differentiates TB patients from healthy controls (AUC value of 0.905).

We and others have confirmed that miRNAs in human serum and plasma are relatively stable [16, 33]. However, the source of circulating miRNAs is still unknown. In a previous study, it was demonstrated that plasma miRNAs were not only derived from circulating blood cells but also from other tissues that were affected by disease [16]. In addition, it has been reported that miRNAs are stored in microvesicles derived from various cell types [16, 33]. This strongly suggests that active secretion by cells is a major source of the miRNAs found in serum and plasma. These findings further support the hypothesis that the miRNA profile in serum and plasma is an indicator of biological function. Extensive studies on miRNA expression patterns in plasma and serum may help to establish an miRNAs profile that is associated with pathological processes in tissues, and evaluate circulating miRNAs that may help understand mechanisms of disease.

It is found for the first time that the expression level of miR-769-5p in TB patients is lower than that in healthy people. miR-769-5p expression has previously been studied in cancer, but its exact role remains unknown [34–39]. Upon reoxygenation of MCF-7 breast cancer cells, miR-769-3p reduces the expression of the N-myc downstream-regulated gene 1, whereas over-expression of miR-769-3p enhances apoptosis [37]. In addition, miR-769-5p, in combination with other miRNAs, is involved in the prognosis of pancreatic cancer and non-small cell lung cancer [35, 38]. The significance of the downregulation of miR-320a in TB patients has not yet been clarified. It has been reported that miR-320a inhibits cell proliferation, migration, and invasion by targeting the BMI-1 gene in nasopharyngeal carcinoma [40], and may be implicated in the α-synuclein aggravation in Parkinson's disease [41]. Importantly, miR-320a plays a role in the modulation of cytokine production [42]. We hypothesized that the decreased expression of miR-320a may facilitate the progression of disease by reactivating cell migration and proliferation in the lung tissue. MiR-22 directly downregulates phosphatase and tensin homolog levels through a specific site on the phosphatase and tensin homolog (PTEN) 3'UTR and acts by fine-tuning the dynamics of the PTEN/AKT/FoxO1 pathway [43]. MDC1 is a critical component of the DNA damage response machinery, and miR-22 impaired DNA damage repair and genomic instability by inhibiting MDC1 translation [44]. In endothelial cells, extracellular uridine triphosphate (UTP) and adenosine triphosphate (ATP) attenuate intercellular adhesion molecule 1 (ICAM-1) expression and leukocyte adhesion through miR-22 [45]. In order to understand the significance of the unique expression pattern of miR-769-5p, miR-320a, and miR-22-3p in TB patients, these miRNAs need to be further investigated, to identify target genes of circulating miRNAs and the mechanism that regulates miRNA biogenesis.

Currently, no circulating miRNAs have been reported that distinguish between patients with and patients without cavity. We measured miRNA levels in plasma derived from TB patients with and without cavity. However, none of the miRNAs evaluated showed significantly different expression between cavity and non-cavity groups. It is indicated that the roles of these miRNAs may be not obvious in affecting the expression of those cell factors during the formation of cavity, and the prognosis differences caused by cavities may be not associated with the expression of these miRNAs. Actually, in the present study, we chose the miRNAs for the next validation that showed at least a 4-fold difference in the expression between the comparative groups in the Solexa sequencing results. To unravel possible mechanisms for cavity formation and to identify potential TB biomarkers, further studies may be required applying a less strict standard.

Drug-resistant TB continues to threaten global TB control and remains a major public health concern in many developing countries. The WHO indicated that in 2015, an estimated 480,000 new cases of MDR-TB and an additional 100,000 cases with rifampicin-resistant TB (RR-TB) were identified. In 2015, China, India, and the Russian Federations accounted for 45% of all MDR/RR-TB cases [1]. In the Heilongjiang Province in China, drug resistance is more severe than in many other areas in China [46]. Global resistance rates to the first-line drugs and MDR-TB were 57.0 and 22.8%, respectively. The primary MDR-TB and pan-resistance rates were as high as 13.6% and 5.0%, respectively [46]. Studies have shown that *M*. *tuberculosis* in MDR patients results in comparatively strong immune responses, resulting in a significant increase in TNF-α and IFN-γ levels in peripheral blood, which play an important role in the pathogenesis of TB [47]. In the present study, miR-320a was significantly downregulated in plasma derived from drug-resistant TB patients compared to drug-susceptible patients. The AUC of miR-320a for drug-resistant and drug-susceptible TB patients was 0.882 (95% CI was 0.80–0.97), implying miR-320a as a potential marker for discriminating between the two conditions. Whether or not miR-320a is associated with drug resistance still needs to be confirmed.

*M*. *tuberculosis* is transmitted through droplet infection, and affects the lives of individuals that are in close contact with TB patients or asymptomatic undiagnosed subjects. Rapid and accurate diagnosis and adequate antimicrobial therapy is critical to control TB spread [48]. Regarding distinguishing TB disease and predicting drug resistance, the novel panel of miR-22, miR-320a, and miR-769-5p and miR-320a will be helpful.

The main limitation of the present study is that no other lung diseases were included as controls for TB. To further validate the three miRNAs in discriminating TB from other lung diseases and to adjudge this panel in TB diagnoses, we will include appropriate lung disease control groups in our future studies.

## Supporting information

S1 Fig Analysis of the length distribution of plasma small RNAs. (A) CP-TB patients, (B) NCP-TB patients and (C) healthy controls.
(TIF)

S2 Fig Scatter plot of miRNA expression in pooled plasma from healthy control, non-cavity patients and cavity patients (control: x; treatment: y). **(A)** CP-TB patients vs. healthy controls; (B) NCP-TB patients vs. healthy controls; (C) NCP-TB patients vs. CP-TB patients.
(TIF)

S1 Table Categories of small RNAs in pooled plasma from healthy controls, non-cavity patients and cavity patients measured by Illumina high-throughput sequencing technology. (DOCX)

S2 Table Differentially-expressed miRNAs in plasma samples from TB patients compared to healthy controls determined by Illumina high-throughput sequencing. (DOCX)

S3 Table 27 differentially-expressed miRNAs determined by Illumina high-throughput sequencing in Biomarker-selection phase. (DOCX)
