# Supplementary figures and images for: Characterization of a novel panel of plasma microRNAs that discriminates between Mycobacterium tuberculosis infection and healthy individuals
Source: PLoS One. 2017 Sep 14;12(9):e0184113. doi: 10.1371/journal.pone.0184113 (PMC5598944; doi:10.1371/journal.pone.0184113)

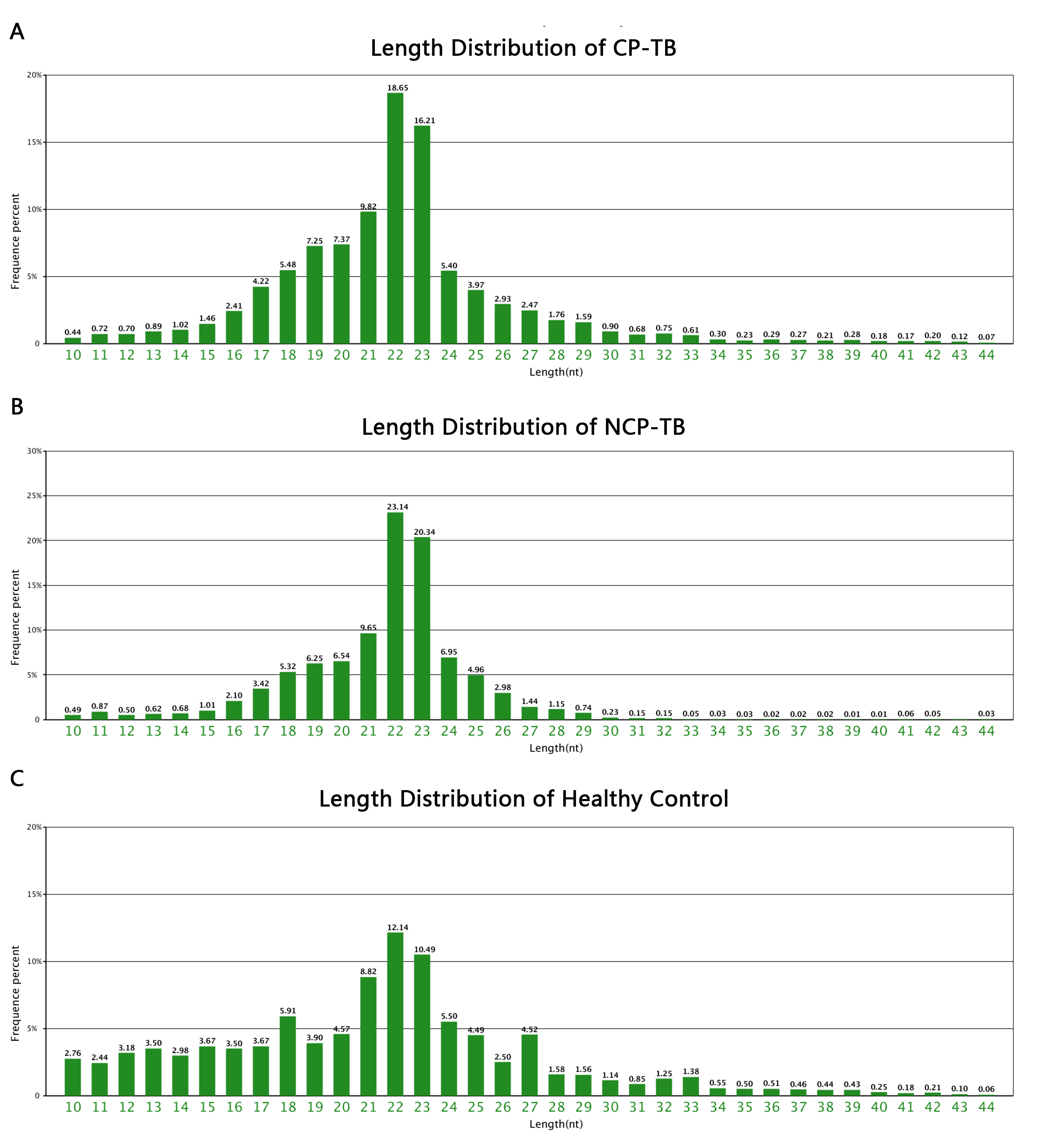

Supplement: S1 Fig — (A) CP-TB patients, (B) NCP-TB patients and (C) healthy controls. (TIF) [file pone.0184113.s001.tif]

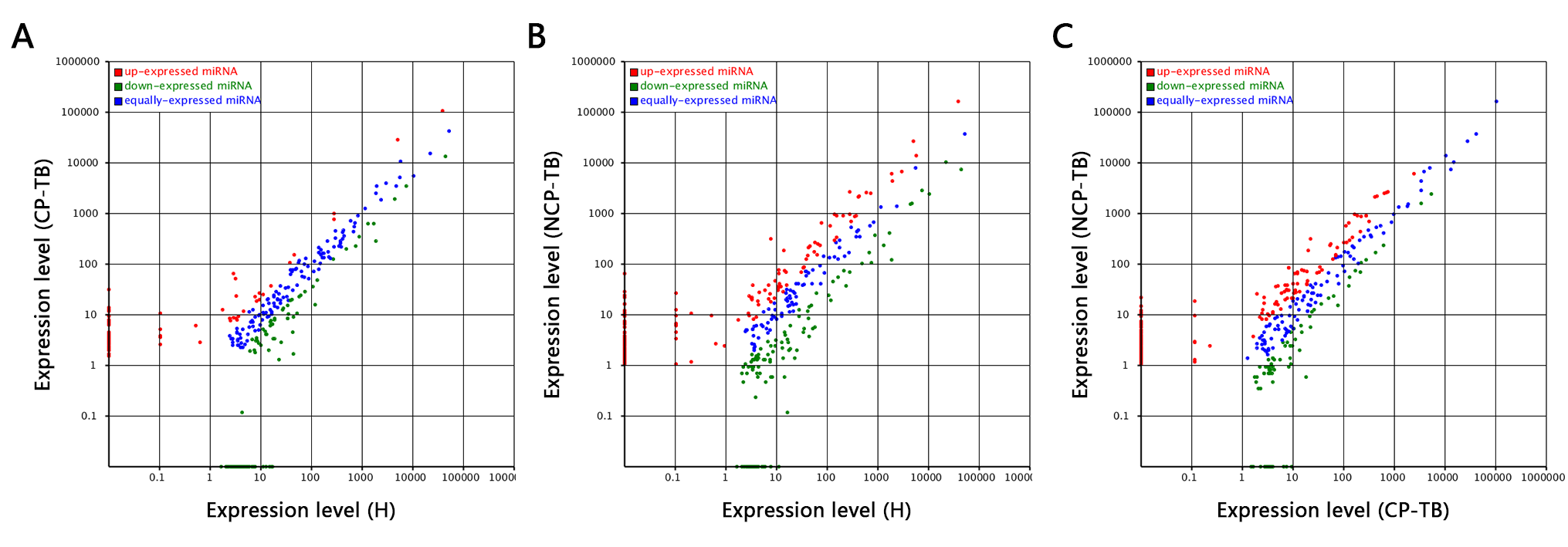

Supplement: S2 Fig — (A) CP-TB patients vs. healthy controls; (B) NCP-TB patients vs. healthy controls; (C) NCP-TB patients vs. CP-TB patients. (TIF) [file pone.0184113.s002.tif]
