# Supplementary material for: Characterization of a novel panel of plasma microRNAs that discriminates between Mycobacterium tuberculosis infection and healthy individuals
Source: PLoS One. 2017 Sep 14;12(9):e0184113. doi: 10.1371/journal.pone.0184113 (PMC5598944; doi:10.1371/journal.pone.0184113)
Supplement: S1 Table — (DOCX) [file pone.0184113.s003.docx]

**Supplemental Table 1. The categories of small RNAs in pooled plasma from healthy controls, non-cavity and cavity patients by Solexa sequencing technology.**

|  | Healthy controls  (10 ml) | NCP-TB  (10 ml) | CP-TB  (10 ml) |
| --- | --- | --- | --- |
| Total  (match genome) | 3155127 | 5118983 | 5270959 |
| miRNA | 23.09% | 31.88% | 25.87% |
| rRNA | 13.58% | 27.94% | 35.83% |
| scRNA | 3.09% | 0.61% | 0.94% |
| snRNA | 0.13% | 1.53% | 1.40% |
| snoRNA | 0.21% | 0.28% | 0.17% |
| srpRNA | 0.00% | 0.01% | 0.01% |
| tRNA | 1.42% | 1.12% | 1.77% |
| repeat | 0.37% | 0.46% | 0.70% |
| Exon-antisense | 0.01% | 0.01% | 0.01% |
| Exon-sense | 0.34% | 0.63% | 0.79% |
| Intron-antisense | 0.07% | 0.02% | 0.06% |
| Intron-sense | 0.16% | 0.11% | 0.18% |
| unannotated | 57.53% | 35.40% | 32.27% |
