# Supplementary material for: Characterization of a novel panel of plasma microRNAs that discriminates between Mycobacterium tuberculosis infection and healthy individuals
Source: PLoS One. 2017 Sep 14;12(9):e0184113. doi: 10.1371/journal.pone.0184113 (PMC5598944; doi:10.1371/journal.pone.0184113)
Supplement: S2 Table — (DOCX) [file pone.0184113.s004.docx]

| miRNA | Copy number in Healthy controls | Copy number in NCP-TB | NCP-TB / Healthy | Copy number in CP-TB | CP-TB / Healthy | CP-TB / NCP-TB |
| --- | --- | --- | --- | --- | --- | --- |
| hsa-miR-16-5p | 74 | 2681 | 36.23 | 194 | 2.62 | 0.07 |
| hsa-let-7d-3p | 133 | 1570 | 11.80 | 171 | 1.29 | 0.11 |
| hsa-miR-148a-3p | 2691 | 22619 | 8.41 | 6444 | 2.39 | 0.28 |
| hsa-miR-26a-5p | 738 | 5612 | 7.60 | 1084 | 1.47 | 0.19 |
| hsa-let-7b-5p | 105 | 661 | 6.30 | 129 | 1.23 | 0.20 |
| hsa-let-7f-5p | 1340 | 8131 | 6.07 | 1425 | 1.06 | 0.18 |
| hsa-miR-21-5p | 1467 | 7654 | 5.22 | 1601 | 1.09 | 0.21 |
| hsa-miR-10b-5p | 48758 | 227534 | 4.67 | 241298 | 4.95 | 1.06 |
| hsa-miR-128-3p | 139 | 636 | 4.58 | 141 | 1.01 | 0.22 |
| hsa-miR-107 | 3957 | 17959 | 4.54 | 3609 | 0.91 | 0.20 |
| hsa-miR-103a-3p | 4152 | 18710 | 4.51 | 3878 | 0.93 | 0.21 |
| hsa-let-7a-5p | 1097 | 4857 | 4.43 | 966 | 0.88 | 0.20 |
| hsa-miR-23a-3p | 411 | 1787 | 4.35 | 653 | 1.59 | 0.37 |
| hsa-miR-100-5p | 443 | 1925 | 4.35 | 1269 | 2.86 | 0.66 |
| hsa-miR-181b-5p | 545 | 2298 | 4.22 | 973 | 1.79 | 0.42 |
| hsa-miR-101-3p | 149 | 596 | 4.00 | 105 | 0.70 | 0.18 |
| hsa-miR-92a-3p | 1969 | 7759 | 3.94 | 2371 | 1.20 | 0.31 |
| hsa-miR-182-5p | 5696 | 21933 | 3.85 | 5944 | 1.04 | 0.27 |
| hsa-miR-486-5p | 371644 | 1384947 | 3.73 | 892295 | 2.40 | 0.64 |
| hsa-miR-130a-3p | 630 | 2115 | 3.36 | 486 | 0.77 | 0.23 |
| hsa-miR-27a-3p | 400 | 1251 | 3.13 | 596 | 1.49 | 0.48 |
| hsa-miR-142-5p | 6911 | 21394 | 3.10 | 5508 | 0.80 | 0.26 |
| hsa-miR-99b-5p | 2701 | 8267 | 3.06 | 8455 | 3.13 | 1.02 |
| hsa-miR-181a-5p | 17941 | 52097 | 2.90 | 21222 | 1.18 | 0.41 |
| hsa-miR-941 | 370 | 1072 | 2.90 | 526 | 1.42 | 0.49 |
| hsa-miR-140-3p | 700 | 2021 | 2.89 | 465 | 0.66 | 0.23 |
| hsa-miR-130b-3p | 535 | 1511 | 2.82 | 909 | 1.70 | 0.60 |
| hsa-miR-4446-3p | 107 | 292 | 2.73 | 214 | 2.00 | 0.73 |
| hsa-miR-24-3p | 332 | 756 | 2.28 | 296 | 0.89 | 0.39 |
| hsa-miR-27b-3p | 3346 | 7444 | 2.22 | 1901 | 0.57 | 0.26 |
| hsa-miR-589-5p | 150 | 331 | 2.21 | 311 | 2.07 | 0.94 |
| hsa-miR-191-5p | 54848 | 119777 | 2.18 | 89839 | 1.64 | 0.75 |
| hsa-miR-126-5p | 3531 | 7604 | 2.15 | 2382 | 0.67 | 0.31 |
| hsa-miR-30d-5p | 2819 | 5984 | 2.12 | 2750 | 0.98 | 0.46 |
| hsa-miR-93-5p | 611 | 1290 | 2.11 | 598 | 0.98 | 0.46 |
| hsa-miR-25-3p | 28303 | 57604 | 2.04 | 33031 | 1.17 | 0.57 |
| hsa-miR-21-3p | 1459 | 2909 | 1.99 | 1279 | 0.88 | 0.44 |
| hsa-miR-99a-5p | 297 | 590 | 1.99 | 286 | 0.96 | 0.48 |
| hsa-miR-10a-5p | 18501 | 36749 | 1.99 | 29008 | 1.57 | 0.79 |
| hsa-miR-19b-3p | 1324 | 2523 | 1.91 | 1762 | 1.33 | 0.70 |
| hsa-miR-425-3p | 131 | 240 | 1.83 | 168 | 1.28 | 0.70 |
| hsa-miR-424-3p | 361 | 619 | 1.71 | 910 | 2.52 | 1.47 |
| hsa-miR-30a-5p | 371 | 602 | 1.62 | 163 | 0.44 | 0.27 |
| hsa-miR-501-3p | 2854 | 4492 | 1.57 | 3810 | 1.33 | 0.85 |
| hsa-let-7i-5p | 1450 | 2247 | 1.55 | 878 | 0.61 | 0.39 |
| hsa-miR-223-5p | 234 | 351 | 1.50 | 164 | 0.70 | 0.47 |
| hsa-miR-30e-3p | 1700 | 2538 | 1.49 | 1135 | 0.67 | 0.45 |
| hsa-miR-425-5p | 812 | 1207 | 1.49 | 759 | 0.93 | 0.63 |
| hsa-miR-375 | 366 | 500 | 1.37 | 649 | 1.77 | 1.30 |
| hsa-miR-363-3p | 415 | 564 | 1.36 | 257 | 0.62 | 0.46 |
| hsa-let-7c-5p | 261 | 351 | 1.34 | 106 | 0.41 | 0.30 |
| hsa-miR-500a-3p | 505 | 657 | 1.30 | 319 | 0.63 | 0.49 |
| hsa-miR-143-3p | 53541 | 68616 | 1.28 | 42834 | 0.80 | 0.62 |
| hsa-miR-652-3p | 207 | 245 | 1.18 | 117 | 0.57 | 0.48 |
| hsa-miR-181c-5p | 175 | 203 | 1.16 | 144 | 0.82 | 0.71 |
| hsa-miR-181a-3p | 701 | 788 | 1.12 | 841 | 1.20 | 1.07 |
| hsa-miR-532-5p | 1661 | 1791 | 1.08 | 1374 | 0.83 | 0.77 |
| hsa-miR-223-3p | 1074 | 1143 | 1.06 | 597 | 0.56 | 0.52 |
| hsa-miR-30a-3p | 216 | 228 | 1.06 | 221 | 1.02 | 0.97 |
| hsa-miR-150-5p | 310 | 324 | 1.05 | 194 | 0.63 | 0.60 |
| hsa-miR-146b-5p | 3658 | 3775 | 1.03 | 1835 | 0.50 | 0.49 |
| hsa-miR-151a-5p | 3899 | 4022 | 1.03 | 2640 | 0.68 | 0.66 |
| hsa-miR-192-5p | 11068 | 11360 | 1.03 | 10575 | 0.96 | 0.93 |
| hsa-miR-145-3p | 332 | 335 | 1.01 | 161 | 0.48 | 0.48 |
| hsa-miR-342-3p | 1338 | 1191 | 0.89 | 659 | 0.49 | 0.55 |
| hsa-miR-451a | 3567 | 2948 | 0.83 | 2147 | 0.60 | 0.73 |
| hsa-miR-199a-5p | 236 | 193 | 0.82 | 143 | 0.61 | 0.74 |
| hsa-miR-125a-5p | 194 | 149 | 0.77 | 240 | 1.24 | 1.61 |
| hsa-miR-4677-3p | 173 | 131 | 0.76 | 191 | 1.10 | 1.46 |
| hsa-miR-221-3p | 6689 | 4930 | 0.74 | 4573 | 0.68 | 0.93 |
| hsa-miR-345-5p | 479 | 350 | 0.73 | 681 | 1.42 | 1.95 |
| hsa-miR-423-3p | 8023 | 5758 | 0.72 | 7691 | 0.96 | 1.34 |
| hsa-miR-106b-3p | 4196 | 2942 | 0.70 | 3070 | 0.73 | 1.04 |
| hsa-miR-222-3p | 1553 | 1082 | 0.70 | 1353 | 0.87 | 1.25 |
| hsa-miR-151b | 853 | 565 | 0.66 | 431 | 0.51 | 0.76 |
| hsa-miR-146b-3p | 207 | 136 | 0.66 | 171 | 0.83 | 1.26 |
| hsa-miR-423-5p | 502946 | 316404 | 0.63 | 358886 | 0.71 | 1.13 |
| hsa-miR-7706 | 234 | 140 | 0.60 | 308 | 1.32 | 2.20 |
| hsa-miR-584-5p | 2124 | 1225 | 0.58 | 1148 | 0.54 | 0.94 |
| hsa-miR-181a-2-3p | 2603 | 1449 | 0.56 | 1062 | 0.41 | 0.73 |
| hsa-miR-28-3p | 22987 | 11968 | 0.52 | 15615 | 0.68 | 1.30 |
| hsa-miR-877-5p | 705 | 348 | 0.49 | 243 | 0.34 | 0.70 |
| hsa-miR-331-3p | 208 | 101 | 0.49 | 167 | 0.80 | 1.65 |
| hsa-miR-22-3p | 211742 | 89038 | 0.42 | 129973 | 0.61 | 1.46 |
| hsa-miR-342-5p | 271 | 106 | 0.39 | 113 | 0.42 | 1.07 |
| hsa-miR-744-5p | 8348 | 3161 | 0.38 | 2916 | 0.35 | 0.92 |
| hsa-miR-181c-3p | 595 | 225 | 0.38 | 203 | 0.34 | 0.90 |
| hsa-miR-146a-5p | 70477 | 24185 | 0.34 | 29054 | 0.41 | 1.20 |
| hsa-miR-7849-3p | 1233 | 386 | 0.31 | 404 | 0.33 | 1.05 |
| hsa-miR-148a-5p | 2037 | 634 | 0.31 | 1479 | 0.73 | 2.33 |
| hsa-miR-186-5p | 42009 | 12982 | 0.31 | 16390 | 0.39 | 1.26 |
| hsa-miR-339-5p | 1565 | 470 | 0.30 | 1137 | 0.73 | 2.42 |
| hsa-miR-320b | 45267 | 13539 | 0.30 | 29104 | 0.64 | 2.15 |
| hsa-miR-4286 | 467 | 132 | 0.28 | 659 | 1.41 | 4.99 |
| hsa-miR-874-3p | 397 | 101 | 0.25 | 170 | 0.43 | 1.68 |
| hsa-miR-127-3p | 6447 | 1455 | 0.23 | 3670 | 0.57 | 2.52 |
| hsa-miR-1307-5p | 2695 | 597 | 0.22 | 1848 | 0.69 | 3.10 |
| hsa-miR-378a-3p | 16220 | 3541 | 0.22 | 5347 | 0.33 | 1.51 |
| hsa-miR-320a | 99787 | 20602 | 0.21 | 46445 | 0.47 | 2.25 |
| hsa-miR-873-3p | 1011 | 205 | 0.20 | 300 | 0.30 | 1.46 |
| hsa-miR-6852-5p | 4705 | 873 | 0.19 | 1686 | 0.36 | 1.93 |
| hsa-miR-409-3p | 12578 | 2012 | 0.16 | 5266 | 0.42 | 2.62 |
| hsa-miR-185-3p | 1128 | 166 | 0.15 | 132 | 0.12 | 0.80 |
| hsa-miR-151a-3p | 427590 | 62279 | 0.15 | 112835 | 0.26 | 1.81 |
| hsa-miR-1307-3p | 2260 | 315 | 0.14 | 1105 | 0.49 | 3.51 |
| hsa-miR-486-3p | 7175 | 899 | 0.13 | 1915 | 0.27 | 2.13 |
| hsa-miR-769-5p | 17706 | 1037 | 0.06 | 2370 | 0.13 | 2.29 |
