# Supplementary material for: Characterization of a novel panel of plasma microRNAs that discriminates between Mycobacterium tuberculosis infection and healthy individuals
Source: PLoS One. 2017 Sep 14;12(9):e0184113. doi: 10.1371/journal.pone.0184113 (PMC5598944; doi:10.1371/journal.pone.0184113)
Supplement: S3 Table — (DOCX) [file pone.0184113.s005.docx]

| miRNA | Copy number in Healthy controls | Copy number in NCP-TB | NCP-TB / Healthy | Copy number in CP-TB | CP-TB / Healthy | CP-TB / NCP-TB |
| --- | --- | --- | --- | --- | --- | --- |
| hsa-miR-16-5p | 74 | 2681 | 36.23 | 194 | 2.62 | 0.07 |
| hsa-miR-148a-3p | 2691 | 22619 | 8.41 | 6444 | 2.39 | 0.28 |
| hsa-miR-26a-5p | 738 | 5612 | 7.60 | 1084 | 1.47 | 0.19 |
| hsa-let-7f-5p | 1340 | 8131 | 6.07 | 1425 | 1.06 | 0.18 |
| hsa-miR-21-5p | 1467 | 7654 | 5.22 | 1601 | 1.09 | 0.21 |
| hsa-miR-10b-5p | 48758 | 227534 | 4.67 | 241298 | 4.95 | 1.06 |
| hsa-miR-107 | 3957 | 17959 | 4.54 | 3609 | 0.91 | 0.20 |
| hsa-miR-103a-3p | 4152 | 18710 | 4.51 | 3878 | 0.93 | 0.21 |
| hsa-miR-92a-3p | 1969 | 7759 | 3.94 | 2371 | 1.20 | 0.31 |
| hsa-miR-182-5p | 5696 | 21933 | 3.85 | 5944 | 1.04 | 0.27 |
| hsa-miR-486-5p | 371644 | 1384947 | 3.73 | 892295 | 2.40 | 0.64 |
| hsa-miR-142-5p | 6911 | 21394 | 3.10 | 5508 | 0.80 | 0.26 |
| hsa-miR-99b-5p | 2701 | 8267 | 3.06 | 8455 | 3.13 | 1.02 |
| hsa-miR-181a-5p | 17941 | 52097 | 2.90 | 21222 | 1.18 | 0.41 |
| hsa-miR-191-5p | 54848 | 119777 | 2.18 | 89839 | 1.64 | 0.75 |
| hsa-miR-126-5p | 3531 | 7604 | 2.15 | 2382 | 0.67 | 0.31 |
| hsa-miR-10a-5p | 18501 | 36749 | 1.99 | 29008 | 1.57 | 0.79 |
| hsa-miR-28-3p | 22987 | 11968 | 0.52 | 15615 | 0.68 | 1.30 |
| hsa-miR-22-3p | 211742 | 89038 | 0.42 | 129973 | 0.61 | 1.46 |
| hsa-miR-146a-5p | 70477 | 24185 | 0.34 | 29054 | 0.41 | 1.20 |
| hsa-miR-186-5p | 42009 | 12982 | 0.31 | 16390 | 0.39 | 1.26 |
| hsa-miR-320b | 45267 | 13539 | 0.30 | 29104 | 0.64 | 2.15 |
| hsa-miR-320a | 99787 | 20602 | 0.21 | 46445 | 0.47 | 2.25 |
| hsa-miR-409-3p | 12578 | 2012 | 0.16 | 5266 | 0.42 | 2.62 |
| hsa-miR-151a-3p | 427590 | 62279 | 0.15 | 112835 | 0.26 | 1.81 |
| hsa-miR-486-3p | 7175 | 899 | 0.13 | 1915 | 0.27 | 2.13 |
| hsa-miR-769-5p | 17706 | 1037 | 0.06 | 2370 | 0.13 | 2.29 |
